# Social Environmental Factors Associated with Depression Among Older Adults in Busan, South Korea: Based on the 2023 Korea Community Health Survey

**DOI:** 10.3390/healthcare13222867

**Published:** 2025-11-11

**Authors:** Yujin Suh, Hyejin Lee, Yumi Yi, Yunji Lee

**Affiliations:** Department of Nursing, Research Institute of Dong-Eui Nursing Science, Dong-eui University, 176 Eomgwang-ro, Busanjin-gu, Busan 47340, Republic of Korea; yugibabe@deu.ac.kr (Y.S.); hyejin.lee@deu.ac.kr (H.L.); yym7493@deu.ac.kr (Y.Y.)

**Keywords:** aged, depression, social environment, physical environment

## Abstract

**Background/Objectives**: This study examined the prevalence of depression among older adults in Busan—the first metropolitan city in South Korea to become a super-aged society—and identified the social environmental factors associated with depression. **Methods**: Using data from the 2023 Korea Community Health Survey (KCHS), 5143 individuals aged 65 and older residing in Busan were analyzed. Depressive symptoms were measured using the PHQ-9, and social environmental factors—including unmet medical needs, satisfaction with the community environment, participation in social activities, and frequency of social contact—were derived from KCHS items. Descriptive statistics and logistic regression were performed using SPSS, version 29.0. **Results**: Participants’ mean age was 73.50 ± 0.11 years, and 54.4% were female. The average depression score was 2.85 ± 0.06, with 3.7% having moderate, 1.2% moderately severe, and 0.4% severe depression. Logistic regression revealed that low healthcare accessibility was significantly associated with higher odds of moderate-to-severe depression (OR = 2.54, 95% CI = 1.78–3.62). Conversely, higher satisfaction with community environment (OR = 0.80, 95% CI = 0.73–0.87) and greater participation in social activities (OR = 0.68, 95% CI = 0.53–0.87) were associated with lower odds of depression. **Conclusions**: Depression in older adults is a multidimensional phenomenon shaped by the complex interplay of individual, health-related, and socio-environmental factors. Region-specific, community-based programs that enhance living environments, expand social participation, improve healthcare access, and promote positive health perceptions are essential. These findings can inform integrated nursing and policy interventions that support healthy aging and enhance older adults’ well-being.

## 1. Introduction

As of 2025, adults aged 65 years and older account for 20.3% of South Korea’s total population, signifying the nation’s entry into a super-aged society [1]. This demographic shift has heightened social interest in the quality of life and health in later life, alongside an urgent need for policies that address the multifaceted challenges of aging. Older adults often face not only age-related declines in physical health, but also economic vulnerability owing to limited income sources and unstable employment opportunities, coupled with a substantial reduction in social relationships. These intersecting vulnerabilities are increasingly being explained through the concept of social exclusion [2]. Moreover, demographic and cultural changes, such as a rise in single-person households among older adults, indicate that many lack the personal and household resources needed to overcome social exclusion independently.

Furthermore, the COVID-19 pandemic has had a profound and lasting impact on mental health, particularly among older adults. Previous studies have documented increases in depressive symptoms, anxiety, and social isolation during and after the pandemic period [3,4]. For older individuals, prolonged physical distancing and disruptions in community activities weakened social networks and reduced access to essential health and welfare services. These changes likely amplified preexisting vulnerabilities associated with aging, highlighting the need to comprehensively explore the social and environmental determinants of depression among older adults in the post-pandemic era.

The combined effects of these challenges can significantly compromise psychological well-being, often leading to mental health conditions, such as depression [5]. According to the 2023 National Survey on Older Adults, 11.3% of individuals aged 65 years and older were at risk for depression [6]. Previous studies have identified a range of associated factors, including demographic characteristics (e.g., gender, age), socioeconomic status (e.g., education, income, occupation), and health status [7,8,9]. In addition, reductions in social engagement and age-related functional decline have been categorized as environmental determinants of late-life depression [5,10,11,12,13]. These environmental factors can be broadly classified into (1) social–environmental aspects, such as social participation, relationship with one’s neighbor, and perceived satisfaction with one’s surroundings; and (2) physical-environmental aspects, including accessibility to healthcare and community facilities, and opportunities for outdoor activity. Furthermore, individual health-related factors, such as chronic illness and functional limitations, may interact with these environmental influences. Therefore, it is essential to identify which environmental elements are most strongly associated with depression in older adults.

Older adults tend to be more dependent on their surrounding environment than younger populations owing to age-related declines in physical functioning and reduced activity levels [12]. In this context, resource-rich communities can serve a compensatory role by offsetting individual capacity losses among older adults.

A growing body of research has emphasized the importance of neighborhood and community environments in shaping the mental health of older adults. Community satisfaction, including perceptions of neighborhood safety, greenery, and housing conditions, has been shown to significantly affect depressive symptoms in later life [14]. In particular, social relationships with neighbors and levels of community cohesion serve as important determinants of emotional well-being among older adults [15,16]. Social participation has consistently been identified as a protective factor against depression, with higher frequency and greater diversity of social engagement associated with lower depressive symptomatology [17]. Likewise, limited opportunities for participation or social isolation contribute to heightened risks of psychological distress in aging populations [18]. Healthcare accessibility, including proximity to medical institutions, availability of transportation, and the financial burden of medical services also plays a critical role in determining mental health outcomes among older adults [19].

Busan is the fastest-aging city in South Korea, with a population aging index of 0.968 [20], and as of 2023, older adults aged 65 years and above accounted for 23.5% of its population, making it the first metropolitan city in the country to become a super-aged society [21]. In addition, Busan presents unique socio-spatial characteristics. Marked disparities exist between affluent coastal districts and more socioeconomically disadvantaged inland or western areas. These disparities are reflected in housing conditions, neighborhood environments, access to healthcare facilities, and transportation infrastructure [22,23,24], which in turn may shape the mental health of older adults. These inequalities in housing, healthcare accessibility, and transportation make Busan an ideal setting for examining how multilevel environmental factors influence late-life mental health. Insights derived from this context can guide community-level policies to improve healthcare accessibility, enhance age-friendly environments, and reduce regional disparities in mental health among older adults in rapidly aging urban settings.

Therefore, this study aimed to investigate the prevalence of depression among adults aged 65 years and older in Busan using 2023 Korea Community Health Survey (KCHS) data provided by the Korea Disease Control and Prevention Agency (KDCA) [25] and to analyze the association between social environmental factors on depression. Specifically, this study addressed the following research questions: (1) What is the prevalence of moderate-to-severe depression among older adults in Busan? (2) How are key social–environmental factors—including unmet medical needs, satisfaction with the social environment, participation in social activities, and frequency of social contact—associated with depression in this population?

## 2. Materials and Methods

### 2.1. Study Design

This descriptive correlational study—a secondary data analysis using raw data from the 2023 KCHS—was conducted to examine the relationship between depression and social environmental factors among adults aged 65 years and older residing in Busan Metropolitan City, South Korea.

### 2.2. Participants and Data Collection

The 2023 KCHS, a nationwide survey administered by the KDCA in accordance with the Regional Public Health Act, employed a two-stage probability proportional stratified sampling method, using probability proportional to size in the first stage, and systematic sampling in the second stage. Trained interviewers conducted face-to-face interviews with adults aged 19 years and older residing in randomly selected households between 16 May and 31 July 2023 [26].

The participants of this study were older adults aged 65 years and older residing in Busan who participated in the 2023 KCHS. Of the 231,752 respondents nationwide, 14,513 resided in Busan, and 5189 of them were aged 65 years or older, meeting the inclusion criteria for this study. Since depression was the primary dependent variable in this study, cases with any missing or “unknown” responses to one or more of the nine depression-related items in the KCHS were excluded, as it was not possible to calculate a total score. A total of 46 such cases (approximately 0.9% of all older adult respondents in Busan) were excluded, resulting in a final sample of 5143 participants.

### 2.3. Measurements

#### 2.3.1. General and Health-Related Characteristics

General characteristics comprised sex, age, marital status (living with versus without a spouse), employment status (engaged versus not engaged in income-generating activities) and receiving basic livelihood security benefits.

Health-related characteristics included smoking status (current smoker of conventional or electronic cigarettes versus non-smoker), alcohol consumption, level of physical activity, number of chronic diseases, fall experiences in the past year, and perceived health status. Physical activity levels were assessed using the Korean version of the International Physical Activity Questionnaire (IPAQ) [27] and categorized as inactive (<600 MET-min/week), minimally active (600–2999 MET-min/week), or health-enhancing physically active (HEPA; ≥3000 MET-min/week). The number of chronic diseases was determined from self-reported physician diagnoses of hypertension and diabetes, with counts ranging from 0 to 2. For IPAQ, cases with “don’t know” or non-response were treated as zero when calculating total physical activity, and no missing values were observed for any other variables.

#### 2.3.2. Social Environmental Factors

Social environmental factors included unmet medical needs, satisfaction with the community environment, participation in social activities, and frequency of social contact. Unmet medical needs were defined as responding “yes” to either of two questions regarding unmet dental or medical care needs in the past year. Since unmet medical needs are associated with both the physical and social accessibility of healthcare services [28,29], they were classified as an indicator of healthcare accessibility and included as one of the variables representing the community environment related to older adults’ mental health.

Satisfaction with the community environment was assessed by summing responses to seven items: trust in neighbors, willingness of neighbors to help during family events (e.g., weddings or funerals), and satisfaction with: community safety, natural environment, residential environment, public transportation, and local healthcare services. Each “yes” response was scored as 1 and “no/uncertain” as 0, yielding a total score range of 0–7.

Participation in social activities was calculated as the number of activity types regularly engaged in at least once a month, including religious activities, social gatherings, leisure or recreational activities, and volunteer work with charitable organizations. Each “yes” response was scored as 1 and “no” as 0, with total score range of 0–3.

The frequency of social contact was measured by the frequency of contact with relatives, neighbors, and friends. Contact once a week or more was scored as 1, and less frequent contact as 0. The three items were summed to yield a total score range of 0–2.

For all social environmental variables, responses of “don’t know” or “no answer” were treated as zero in the calculation.

#### 2.3.3. Depression

Depression was assessed using the Korean version of the Patient Health Questionnaire-9 (PHQ-9), a nine-item scale based on the Diagnostic and Statistical Manual of Mental Disorders, Fourth Edition (DSM-IV) diagnostic criteria for major depressive disorders, and employed in KCHS to screen for depressive symptoms [30]. Each item was rated on a 4-point Likert scale (0 = “not at all,” 1 = “several days,” 2 = “more than half the days,” and 3 = “nearly every day,”), yielding a total score range of 0–27. Depression severity was categorized as normal (0–4), mild (5–9), moderate (10–14), moderately severe (15–19), and severe (20–27). In this study, a PHQ-9 score of 10 or higher—corresponding to moderate-to-severe depression—was classified as indicative of depression [30,31]. In a previous study using the Korean version of the PHQ-9 among older adults, the tool demonstrated good reliability with a Cronbach’s α of 0.86 [31]. In this study, the internal consistency of the PHQ-9 was also acceptable, with a Cronbach’s α of 0.79.

### 2.4. Ethical Considerations

This study received approval for exemption from review by the Institutional Review Board (IRB) of Dong-Eui University (DIRB-202502-HR-W-03) and was conducted in compliance with the KDCA guidelines for the use of KCHS raw data, that were obtained from the KCHS website (http://chs.kdca.go.kr) [25] after submitting a formal request and receiving approval through the required review process. All personally identifiable information was removed, and the dataset was provided in a de-identified format with unique identification codes to ensure anonymity.

### 2.5. Data Analysis

All statistical analyses were performed using SPSS Statistics version 29.0 (IBM Corp., Armonk, NY, USA). A two-tailed test with a significance level of 0.05 was applied. Given the complex sampling design of the KCHS, stratification, clustering, and sampling weights were incorporated in all the analyses. Descriptive statistics summarized participants’ characteristics, with unweighted frequencies and weighted percentages, means, and standard errors calculated accordingly. Differences by depression status were examined using Complex–Sample *t*-tests and the Rao-Scott adjusted chi-square test. Factors associated with depression were identified through complex sample logistic regression analysis. Residual independence as well as multicollinearity among the independent variables were assessed using standard multiple linear regression analysis.

## 3. Results

### 3.1. Sociodemographic and Health-Related Characteristics

The sociodemographic and health-related characteristics of the participants are presented in Table 1. The mean age was 73.5 years, with the majority (60.1%) aged 65–74 years, and most participants (65.2%) living with a spouse. Only 29.2% were engaged in economic activities, and 10.7% were recipients of the National Basic Livelihood Security Program, indicating a considerable degree of economic vulnerability among older adults in Busan.

Regarding health behaviors and conditions, 10.2% were current smokers and 45.6% consumed alcohol, of whom 12.0% drank more than twice per week. More than half of the participants (52.2%) were minimally active. Approximately 85% had at least one chronic disease, and 17% had experienced a fall in the past year, reflecting a high likelihood of physical health burdens and functional decline. Moreover, about one-third of participants perceived their overall health as “poor” or “very poor”, suggesting that a substantial proportion of this population experiences both objective and subjective health challenges.

### 3.2. Level of Depression and Social Environmental Factors in Participants

The mean depression score was 2.85 (SE = 0.06), and 5.3% (n = 216) met the diagnostic criteria for moderate-to-severe depression (PHQ-9 score ≥10) (Table 2). Although this percentage appears modest, it indicates that approximately one in twenty older adults in Busan experience clinically relevant depressive symptoms, suggesting elevated vulnerability to mental health risks in this population.

As shown in Table 3, 15.4% of participants reported unmet medical needs in the past year, with the proportion rising to 35.7% among those with moderate-to-severe depression. Participants with moderate-to-severe depression also reported significantly lower levels of satisfaction with the social environment, participation in social activities, and frequency of social contact compared with those without or with mild depression (all *p* < 0.001).

### 3.3. Factors Associated with the Occurrence of Depression in Participants

Table 4 presents the results of Complex–Sample logistic regression analysis. In Model I, unmet medical needs, satisfaction with the social environment, and participation in social activities were significantly associated with depression, whereas the frequency of social contact was not. Participants with unmet medical needs had 2.83 times higher odds of experiencing depression (*p* < 0.001). Each one-point increase in satisfaction with the social environment and participation in social activities was associated with 22% (OR = 0.78, *p* < 0.001) and 48% (OR = 0.52, *p* < 0.001) lower odds of depression, respectively.

In Model II, which adjusted for sociodemographic and health-related variables, these three factors remained significant. Older adults with unmet medical needs had 2.54 times higher odds of experiencing depression (*p* < 0.001), suggesting that limited access to healthcare services represents the strongest social environmental predictor of late-life depression. Each one-point increase in satisfaction with the social environment and participation in social activities was associated with 20% (OR = 0.80, *p* < 0.001) and 32% (OR = 0.68, *p* = 0.002) lower odds of depression, respectively, indicating their protective effects against depressive symptoms.

Among sociodemographic variables, being female (*p* = 0.002), aged 75–84 years compared with 65–74 years (*p* < 0.001), and engaging in economic activity (*p* = 0.001) were associated with higher odds of depression. Among health-related factors, current smoking (*p* = 0.002), experiencing a fall in the past year (*p* < 0.001), and poor perceived health status (*p* < 0.001) were significantly associated with higher odds, whereas alcohol consumption was associated with lower odds. Among all factors, poor perceived health status had the strongest association with depression. Health-related variables, such as unmet medical needs and perceived health status, were strongly associated with depression among older adults, highlighting the critical influence of healthcare accessibility and overall health perception on mental well-being.

No multicollinearity issues were detected (VIF = 1.03–1.48; tolerance = 0.67–0.97). The explanatory power (Nagelkerke R^2^) of Model II was 21.6%.

## 4. Discussion

This study was conducted to examine the association between moderate-to-severe depression and social environmental factors among older adults aged 65 years or older in Busan, South Korea, using raw data from the 2023 KCHS. Older adults residing in Busan showed a relatively higher prevalence of depression compared to the national average, and depression was found to be significantly associated with key social environmental factors, including unmet medical needs, satisfaction with the social environment, and participation in social activities.

The participants’ mean age was 73.5 years, with those aged 65–74 years accounting for the largest proportion at 60.1%, similar to the national distribution of younger-old adults (59.1%) reported by Statistics Korea in 2023 [32]. The proportion of those living with a spouse (65.2%) was higher than the national average (55.2%), whereas the economic activity rate (29.2%) was lower than the national average (39.0%) [32]. Economic activity is not only a means of financial stability for older adults, but also contributes to maintaining social roles [33], enhancing self-esteem [33], and promoting psychological well-being [8]. The low level of economic activity observed among participants suggests that they may face increased needs for financial support and potential psychological risks, such as social isolation and diminished self-esteem, resulting from reduced social engagement. Furthermore, 10.7% of older adults in Busan were recipients of the National Basic Livelihood Security Program, exceeding both the national rate (4.8%) [34] and the range reported in other regions (4.5–9.0%) [35], indicating that older adults in Busan are more likely to experience economic hardship in real terms.

Among participants, 85.0% had at least one chronic condition, such as hypertension or diabetes, which was similar to the national average (86.1%) [34]. The proportion of those who experienced a fall in the past year (17.0%) was higher than the national rate of 14.8% [36]. Multiple chronic conditions and fall experiences can lead to reduced physical functioning and activity, while increased physical discomfort, psychological stress, and fear of recurrence may contribute to emotional distress and diminished quality of life [37,38,39]. Therefore, sustained attention and preventive strategies are needed to promote physical health and maintain functional ability among older adults.

The mean depression score among participants was 2.85 (SE = 0.06), and 5.3% met the criterion for moderate-to-severe depression (PHQ-9 ≥ 10). This prevalence remains higher than the national average (4.1%) [36], indicating a meaningful mental health concern among older adults in Busan. Given the considerable regional variation in depression prevalence (ranging from 0.6% in Gyeryong, Chungnam, to 8.2% in Sasang, Busan) [36], this finding underscores the importance of region-specific approaches to address mental health disparities in rapidly aging urban populations. In particular, concerted efforts by healthcare professionals are needed in cities like Busan, where distinctive social and spatial characteristics may shape depression patterns, emphasizing the importance of contextual analyses to identify region-specific vulnerabilities among older adults.

A particularly notable finding was the high rate of unmet medical needs (15.4%), which was more than twice that of the general population (6.0%) [40]. The relationship between unmet medical needs and depression was also pronounced—35.7% of participants with moderate-to-severe depression reported unmet medical needs, over twice the proportion of those without depression. Previous studies have suggested that when essential medical services for maintaining physical health are not adequately met, emotional problems such as depression may arise [19,29]. Conversely, depression can also reduce healthcare-seeking behavior, treatment adherence, and self-management [41], thereby exacerbating physical health conditions. These interlinked factors highlight the need for community-based health management services that can address both physical and mental health simultaneously.

Social environmental factors also showed meaningful relationships with depression. Participants with moderate-to-severe depression reported lower satisfaction with their social environment (mean score; 4.7 vs. 5.5), lower participation in social activities (0.6 vs. 1.1), and less frequent social contact (1.3 vs. 1.5) compared to those with no depression or mild depression. These findings align with the Social Cognitive Theory [42], which posits that negative cognitive appraisals of one’s environment can intensify depressive symptoms. Prior studies have demonstrated that higher satisfaction with social environments, stronger community cohesion, and greater social engagement protect against depression by fostering belonging and self-efficacy [15,43,44]. Given the natural reduction in social networks in later life due to retirement, loss of peers, and family changes [45], it is necessary to explore strategies that enhance social participation and improve living environments for older adults. Moreover, since depression is closely related to affective temperament [46], a multidimensional approach that simultaneously addresses both personal and social vulnerabilities is required.

The logistic regression results identified unmet medical needs, satisfaction with the social environment, and participation in social activities as significant factors associated with depression, even after adjusting for sociodemographic and health-related factors. Among these factors, unmet medical needs showed the strongest association with depression, aligning with previous findings from Eimontas et al. [47] and Kim et al. [29]. Unmet medical needs are known to arise from multiple factors, including economic hardship, health status, limited transportation accessibility, and the physical distance to medical facilities [29,41,45,47,48,49]. As such, unmet medical needs are not merely indicators of healthcare utilization but represent a critical dimension of socio-physical environmental vulnerability. The findings of this study suggest that weaknesses in the socio-physical environment may exacerbate emotional problems such as depression among older adults. Therefore, in the context of a rapidly aging society, comprehensive efforts are required to examine the multifaceted aspects of older adults’ living environments, mitigate environmental vulnerabilities, and reduce health inequities.

This study also confirmed that higher satisfaction with the social environment and active participation in social activities were significantly associated with lower levels of depression. Consistent with previous findings, older adults with greater residential satisfaction tend to experience fewer depressive symptoms [50], and participation in one or more social activities substantially reduces the risk of depression [51]. These results can be understood through Lawton and Nahemow’s [52] “environmental press” concept, which suggests that a supportive and positive living environment enhances autonomy and competence, thereby reducing stress and promoting psychological well-being. Furthermore, emotional support obtained through social interactions may act as a key mechanism underlying this protective association [50,53]. Given the strong relationship between depression and the interactive characteristics of community environments, it is essential to develop and implement community-based programs that promote social participation among older adults. Furthermore, to enhance the effectiveness and sustainability of such programs, community environment improvement projects and infrastructure development initiatives should be implemented in parallel.

Perceived health status showed the strongest association with depression. Specifically, older adults who rated their health as “poor” had approximately 6.3 times higher odds of experiencing depression compared to those who rated their health as “good.” This finding is similar to the results of Park et al. [54] from the Korean Elderly Survey (OR = 4.29) and Jun and Jeong [55] from the 2020 KCHS. These results suggest that perceived health status, a concept encompassing not only the presence or absence of disease, but also physical, psychological, and social well-being, has a direct and multifaceted association with depression. Although perceived poor health showed a relatively strong association with depression in this study, the result should be interpreted with caution. The magnitude of this relationship may reflect the combined influence of multiple unmeasured psychosocial and physical factors rather than a straightforward causal link. Moreover, since the overall explanatory power of the model was moderate (approximately 21%), it is likely that additional contextual or individual-level determinants of depression were not fully captured, highlighting the need for further, more comprehensive investigations.

As perceived health status reflects a combination of factors, including functional limitations, pain, fatigue, psychological stress, and lack of social support. Therefore, a multidimensional approach is required to improve it. Specific strategies may include tailored health education programs to bridge the gap between actual and perceived health, community-based regular assessments that monitor both perceived health status and depressive symptoms for early intervention, and the establishment of peer-support groups or volunteer networks to strengthen emotional and social resources.

Overall, this study demonstrates that depression among older adults in Busan is closely related to social–environmental conditions, health status, and economic vulnerability. These findings highlight the need for integrated, multi-level interventions to prevent depression in later life. Given Busan’s distinct socio-spatial characteristics—such as steep terrain, substantial inter-district disparities in transportation accessibility, and uneven distribution of community infrastructure—region-specific strategies are needed to address these contextual challenges. In particular, community-based initiatives that simultaneously improve environmental conditions and expand opportunities for social participation could play an important role in enhancing the well-being of older adults. Further analyses of district-level variations within Busan are also warranted, to enable collaboration between local communities and healthcare professionals in developing tailored, integrated approaches to managing the physical, social, and emotional health of older adults. Such place-based perspectives may ultimately contribute to improving the overall mental health and quality of life of the elderly population in Busan.

Although these findings provide valuable insights into the multidimensional characteristics of late-life depression, the cross-sectional design inherently limits causal interpretation. Therefore, the results should be viewed as preliminary evidence to inform future research and the development of context-specific intervention rather than as direct basis for policy implementation. Nevertheless, this study has notable strengths: it utilized a large, population-based sample from the 2023 KCHS and employed standardized, validated instruments to ensure methodological rigor. Moreover, by concurrently analyzing social–environmental and health-related factors, the study offers a multidimensional framework for understanding depression among older adults and provides baseline data that may guide future strategies to promote mental health in rapidly aging urban regions.

However, these results should be interpreted with caution due to several limitations discussed below. First, because this study employed a cross-sectional and descriptive design, it was not possible to establish causal relationships between depression and social or environmental factors. Future research should adopt longitudinal or experimental designs to clarify the temporal and directional effects of social–environmental factors on depression among older adults. Second, although this study utilized large-scale data from the 2023 KCHS to enhance statistical reliability, it was confined to a single metropolitan area (Busan). Therefore, the findings may not be generalizable to other urban or rural regions with different socio-cultural and environmental characteristics. In addition, as perceptions of healthcare accessibility and social environments may differ across countries, caution should be exercised when extrapolating these findings beyond South Korea. Third, the social–environmental variables used in this study were limited to those included in the KCHS and were analyzed in additive or dichotomous forms. Consequently, they may not fully capture the multidimensional and structural characteristics of community environments, such as the physical environment, social capital, and local support systems. Fourth, because this study relied on self-reported data, the results may have been affected by recall bias, misclassification, or social desirability bias. Although multiple covariates were included to control for confounding, unmeasured contextual factors—such as income inequality, quality of social support, and neighborhood-level deprivation—may still have influenced the observed associations. This suggests that other unmeasured variables could contribute to late-life depression, and future studies should incorporate a broader range of socioeconomic, environmental, and psychological factors to provide a more comprehensive understanding of the issue.

## 5. Conclusions

This study identified depression among older adults in Busan as a multidimensional phenomenon resulting from complex interactions between individual characteristics, health-related factors, and social–environmental conditions. Elucidating these relationships provides an advanced understanding of depression in older adults and offers foundational evidence for the development of integrated nursing interventions and policy strategies aimed at promoting mental health in this population. These findings underscore the need to foster supportive residential environments, expand opportunities for social participation, improve healthcare accessibility, and enhance perceived health status. Despite the limitations related to its cross-sectional design and focus on a single metropolitan area, this study contributes valuable insights into the interplay between the social and health determinants of depression in later life. Future research should employ longitudinal and multi-regional comparative approaches as well as practical evaluations of culturally tailored integrated interventions.

## Figures and Tables

**Table 1:** General and Health-related Characteristics (*N* = 5143).

Characteristics	Categories	n ^†^ (Weighted%) ^‡^
Sex	Male	2180 (45.6)
	Female	2963 (54.4)
Age (Year)	65–74	2998 (60.1)
	75–84	1778 (33.1)
	≥85	367 (6.8)
	M ± SE ^‡^	73.50 ± 0.11
Marital status	With spouse	3190 (65.2)
	Without spouse	1953 (34.8)
Economic activity status	Yes	1529 (29.2)
	No	3614 (70.8)
National Basic Livelihood Security	Yes	634 (10.7)
	No	4509 (89.3)
Smoking	Yes	533 (10.2)
	No	4610 (89.8)
Alcohol consumption	None	2913 (54.4)
	≤once a week	1635 (33.4)
	≥twice a week	595 (12.2)
Physical activity	Inactive	1900 (35.8)
(METs min/week)	Minimally active	2645 (52.2)
	HEPA	598 (12.0)
Chronic diseases (numbers)	0	818 (15.1)
	1	2256 (43.6)
	2	2069 (41.3)
Fall (for last year)	Yes	883 (17.1)
	No	4260 (82.9)
Perceived health status	Better	97 (1.9)
	Good	1021 (20.8)
	Moderate	2232 (43.8)
	Poor	1392 (25.7)
	Very poor	401 (7.8)

HEPA = health-enhancing physically active; M = Mean; METs = metabolic equivalents of task; SE = Standard error, ^†^ non-weighted statistics; ^‡^ weighted estimates.

**Table 2:** Depression Status in Participants (*N* = 5143).

Variables	M ± SE ^‡^ or *n* ^†^ (Weighted% ^‡^)	Min–Max ^†^	Median (IQR) ^†^
Depression	2.85 ± 0.06	0–27	2(0–4)
Normal	4.053 (78.1)		
Mild	829 (16.6)		
Moderate	183 (3.7)		
Moderately severe	60 (1.2)		
Severe	18 (0.4)		

M = mean; Max = Maximum; Min = minimum; SE = standard error, ^†^ non-weighted statistics; ^‡^ weighted estimates.

**Table 3:** Social Environmental Factors and Depressive Disorder in Participations (*N* = 5143).

Variables	Overall	Depressive Disorder			
	N ^†^ (Weighted% ^‡^)	No	Yes	$t\;\mathrm{or}\;\chi^{2}$	*p*
		*n* ^†^ (Weighted % ^‡^)			
Unmet medical services					
No	4396 (84.6)	4225 (85.7)	171 (64.3)	64.92	<0.001
Yes	747 (15.4)	657 (14.3)	90 (35.7)		
Environmental satisfaction					
0	49 (1.0)	42 (0.9)	7 (2.6)	11.44	<0.001
1	58 (1.0)	51 (1.0)	7 (1.6)		
2	145 (2.6)	132 (2.5)	13 (4.2)		
3	290 (5.4)	254 (4.9)	36 (14.1)		
4	577 (11.3)	532 (10.9)	45 (18.5)		
5	1187 (23.7)	1122 (23.5)	65 (26.3)		
6	1407 (28.5)	1358 (29.0)	49 (19.3)		
7	1430 (26.6)	1391 (27.3)	39 (13.4)		
M ± SE ^‡^	5.43 ± 0.02	5.47 ± 0.03	4.68 ± 0.11	7.22	<0.001
Participation in Social activities					
0	1763 (33.4)	1606 (31.8)	157 (61.8)	21.14	<0.001
1	1935 (37.4)	1863 (38.1)	72 (25.7)		
2	1044 (20.7)	1018 (21.3)	26 (9.6)		
3	333 (7.1)	331 (7.4)	2 (1.0)		
4	68 (1.4)	64 (1.4)	4 (1.9)		
M ± SE ^‡^	1.06 ± 0.02	1.09 ± 0.02	0.55 ± 0.06	8.31	<0.001
Frequency of social contact					
0	864 (17.5)	801 (17.2)	63 (22.9)	3.63	0.013
1	1590 (31.6)	1498 (31.3)	92 (36.3)		
2	1673 (32.4)	1603 (32.7)	70 (28.1)		
3	1016 (18.5)	980 (18.8)	36 (12.7)		
M ± SE ^‡^	1.52 ± 0.02	1.53 ± 0.02	1.31 ± 0.06	3.37	<0.001

M = mean; Max = Maximum; Min = minimum; SE = standard error, ^†^ non-weighted statistics; ^‡^ weighted estimates.

**Table 4:** Social Environmental Factors Associated with Depression (N = 5143).

Variables		Depressive Disorder (OR (95% CI)) ^†^	
		Model I	Model II
Unmet medical services		2.83 ** (2.05–3.91)	2.54 ** (1.78–3.62)
Environmental satisfaction		0.78 ** (0.72–0.85)	0.80 ** (0.73–0.87)
Participation in Social activities		0.52 ** (0.41–0.66)	0.68 * (0.53–0.87)
Frequency of social contact		0.99 (0.86–1.15)	0.94 (0.81–1.09)
Sex	Male (Ref)		
	Female		1.74 ** (1.22–2.50)
Age	65–74 (Ref)		
	75–84		1.77 ** (1.28–2.44)
	≥85		1.35 (0.79–2.31)
Marital status	With spouse (Ref)		
	Without spouse		1.21 (0.88–1.66)
Economic activity status	Yes (Ref)		
	No		1.85 ** (1.28–2.68)
National basic	No (Ref)		
livelihood security	Yes		1.11 (0.77–1.60)
Smoking	No (Ref)		
	Yes		1.92 ** (1.26–2.92)
Alcohol consumption	None (Ref)		
	≤Once a week		0.63 * (0.42–0.93)
	≥Twice a week		0.44 ** (0.21–0.90)
Physical activity	HEPA (Ref)		
(METs min/week)	Minimally active		0.39 ** (0.23–0.67)
	Inactive		0.64 (0.36–1.13)
Chronic diseases	0 (Ref)		
	1		0.79 (0.55–1.13)
	2		0.83 (0.57–1.22)
Fall	No (Ref)		
	Yes		1.85 ** (1.33–2.57)
Perceived health status	Good (Ref)		
	Poor		6.29 ** (2.97–13.34)

CI = confidence interval; HEPA = health-enhancing physically active; METs = metabolic equivalents of task; Ref = reference category; OR = odds ratio, ^†^ weighted logistic regression; * *p* < 0.05; ** *p* < 0.01.

## Data Availability

The dataset used in this study is publicly available from the Korea Disease Control and Prevention Agency (KDCA) through the Korea Community Health Survey (KCHS) website (https://chs.kdca.go.kr/chs/index.do) (accessed on 15 February 2025). Researchers may access and download the data after completing the required registration process.
